# Lidocaine–Liposomes—A Promising Frontier for Transdermal Pain Management

**DOI:** 10.3390/jcm13010271

**Published:** 2024-01-03

**Authors:** Maria Magdalena Leon, Alexandra Maștaleru, Andra Oancea, Teodora Alexa-Stratulat, Cătălina Anișoara Peptu, Bogdan-Ionel Tamba, Valeria Harabagiu, Cristina Grosu, Anisia Iuliana Alexa, Elena Cojocaru

**Affiliations:** 1 Department of Medical Specialties I, “Grigore T. Popa” University of Medicine and Pharmacy, 700115 Iaşi, Romania; leon_mariamagdalena@yahoo.com; 2 Department of Medical Oncology–Radiotherapy, Faculty of Medicine, “Grigore T. Popa” University of Medicine and Pharmacy, 700115 Iaşi, Romania; teodora_alexa@yahoo.com; 3 Department of Natural and Synthetic Polymers, Faculty of Chemical Engineering and Environmental Protection, “Gheorghe Asachi” Technical University of Iasi, 700050 Iasi, Romania; catipeptu@yahoo.co.uk; 4 CEMEX Laboratory, “Grigore T. Popa” University of Medicine and Pharmacy, 700259 Iaşi, Romania; bogdan.tamba@umfiasi.ro; 5 “Petru Poni” Institute of Macromolecular Chemistry, 700487 Iaşi, Romania; hvaleria@icmpp.ro; 6 Department of Medical Specialties III, “Grigore T. Popa” University of Medicine and Pharmacy, 700115 Iaşi, Romania; fcristina_ro@yahoo.com; 7 Department of Surgery II, Discipline of Ophthalmology, “Grigore T. Popa” University of Medicine and Pharmacy, 700115 Iasi, Romania; alexa_anisia@yahoo.com; 8 Department of Morphofunctional Sciences I, “Grigore T. Popa” University of Medicine and Pharmacy, 700115 Iaşi, Romania; ellacojocaru@yahoo.com

**Keywords:** topical analgesic, lidocaine, drug carrier, controlled release, drug delivery

## Abstract

(1) Background: We aim to develop novel gel formulations for transdermal drug delivery systems in acute and inflammatory pain therapy. (2) Methods: We induced inflammation by the injection of λ-carrageenan on the hind paw of 80 Wistar male rats. The animals were randomized into eight groups of 10 rats each: C (placebo gel), E (EMLA^TM^), L (lidocaine 2%), L-CD (lidocaine + cyclodextrin 2.5%), L-LP (lidocaine + liposomes 1.7%), L-CS (lidocaine + chitosan 4%), L-CSh (lidocaine + chitosan hydrochloride), and L-CS-LP (lidocaine + chitosan + liposomes). The behavior response was determined with a hot plate, cold plate, and algesimeter, each being performed at 30, 60, 120, 180, and 240 min after pain induction. At the end of the experiment, tissue samples were collected for histological assessment. (3) Results: L-LP had the greatest anesthetic effects, which was proven on the cold plate test compared to placebo and EMLA^TM^ (all *p* ≤ 0.001). L-CS-LP had a significant effect on cold plate evaluation compared to placebo (*p* ≤ 0.001) and on hot plate evaluation compared to EMLA^TM^ (*p* = 0.018). (4) Conclusions: L-LP is a new substance with a substantial analgesic effect demonstrated by the cold plate in the first 120 min. Further studies with more animals are needed to determine the maximum doses that can be applied for a better analgesia with minimum side effects.

## 1. Introduction

Pain represents a substantial burden to patients, employers, healthcare systems, and society in general [1], continuing to defy health professionals despite all the technological advances and ready-established medical treatments. Its therapy is still a challenge, partly because of several aspects involved in the pathogenesis, such as nociception, emotional, and behavioral factors [2]. The development of topical preparations aims to improve the patient’s compliance with medical treatment, providing effective pain relief, such as the inflammatory pain induced by the administration of λ-carrageenan, with fewer side effects on the central nervous system and a minimal burden of medication [3].

For invasive interventions, in order to control the pain, opioid derivatives are needed. Analgesic substances/NSAIDs do not have the potential to allow for the pain-free performance of maneuvers such as sternal puncture or arterial blood collection. One pharmacological option for pain treatment is the topical application of lidocaine, a local anesthetic of the amino amide type whose mechanism of action is the blockage of the voltage-gated sodium channels [4]. Structurally, lidocaine consists of three components: a lipophilic aromatic ring, intermediate amide chain, and hydrophilic terminal amine. The lipophilic component makes diffusion possible through the membrane, being directly proportional to the anesthetic potency. The presence of the two amine parts in the structure of lidocaine makes it less lipophilic, thus being a molecule with reduced penetrability through intact skin [5].

Several systems have been proposed to increase the permeability of lidocaine, those currently available being iontophoresis [6] and skin pretreatment with laser or low-frequency ultrasound [7]. Given that these methods require special equipment, an alternative that is frequently used is the incorporation of lidocaine into liposomes [8]. In addition to increasing skin penetration, the lidocaine–liposome formula offers other benefits, such as lowering the effective dose, prolonging the duration of action, and lowering systemic toxicity [9]. Thus, lidocaine has been proposed for transdermal administration; today, there exists a wide range of forms of administration: patches, creams, gels, ointments, sprays, and lotions [10]. Solid forms of administration are partially absorbed at the level of intact skin, while eutectic mixtures melt at lower temperatures than the isolated components, thus ensuring higher drug concentrations. For this reason, several formulas that include liposomes, nanovehicles, iontophoresis, or skin needling have been used. Liposomes are colloidal particles formed as concentric bimolecular layers that are capable of encapsulating drugs. They are lipid vesicles that fully enclose an aqueous volume. These lipid molecules are usually phospholipids with or without some additives [11].

During the last few years, many polymers have been tested. Chitosan, the deacetylated derivative of chitin, acts as a penetration enhancer by opening the tight junctions of the epithelium. Chitosan has many properties, including biocompatibility, biodegradability, non-toxicity, and antimicrobial activity, that have attracted much scientific interest [12]. Numerous research studies have indicated that chitosan and its derivatives greatly improve drug absorption through the skin. As a result, various chitosan-based systems for transdermal delivery have been created. For example, chitosan-based porous patches were developed for transdermal delivery [13]; stable chitosan–phospholipid nanofibers have been prepared by electrospinning, demonstrating their efficiency in transdermal delivery [14]; 5-fluorouracil-loaded chitosan nanoparticles with a microwave synergetic effect for transdermal delivery have been recently studied [15]. 

Another group of well-studied enhancers is the liposomes, which are colloidal particles formed as concentric bimolecular layers that are capable of encapsulating drugs [16]. Liposomes penetrate the stratum corneum by adhering onto the skin’s surface, subsequently destabilizing and fusing or mixing with the lipid matrix [17]. Many liposomal formulations based on plain lipids or combinations with other compounds (generally polymers) have been developed for the transdermal delivery of various drugs [18,19,20].

The inclusion of the drugs into cyclodextrins has been used to enhance aqueous solubility and drug stability [21]. The ring has a hydrophilic exterior and lipophilic inner cavity, in which appropriate organic molecules can be entrapped into non-covalent inclusion complexes, leading to increased aqueous solubility and chemical stability. For example, by employing chitosan, a natural polycationic copolymer consisting of glucosamine and N-acetylglucosamine units, a novel anionic polymer (chitosan–EDTA) was generated by the covalent attachment of EDTA to chitosan. Among the different chitosan vehicles, chitosan–laurate and chitosan–myristate hydrogels enhanced lyophilized drug diffusion through the skin with respect to chitosan–palmitate and chitosan–stearate hydrogels. This could be explained by the interaction of the hydrogels with the stratum corneum, increasing the solubility of the drug in the skin [22].

Topical analgesics are a challenge in the treatment of pain. With the help of a new formula, we are trying to identify a new product with superior efficacy, longer duration of action, and superior analgesia, compared to the only worldwide available topical anesthetic for intact skin, which is EMLA^TM^ (Eutectic Mixture of Local Anesthetics; AstraZeneca Ltd., London, UK). The effect is limited to minutes and requires re-administration if analgesia needs to be prolonged. It is known that EMLA can have side effects such as methemoglobinemia, central nervous system toxicity, and cardiotoxicity and cannot be administered in cases of abrasions or open wounds [23]. The lipidic layer of liposomes provides the barrier for drug diffusion, and hence it provides an extended drug release profile.

The primary outcome was to evaluate the anesthetic effect of lidocaine in combination with various components, including chitosan, liposomes, and cyclodextrins, compared to placebo and EMLA^TM^. The withdrawal threshold (WT) was used as the global outcome assessment tool. The human endpoint established for the study was to develop novel gel formulations for transdermal drug delivery systems based on the investigated drugs. We hypothesized that the penetration enhancers used in this trial will control and modulate the drug delivery profile and improve acute pain management. Secondary outcomes were to evaluate the degree of tissue penetration and to assess the negative effects of the formulas applied, including histological inflammation and local necrosis.

## 2. Materials and Methods

**Figure 1:**
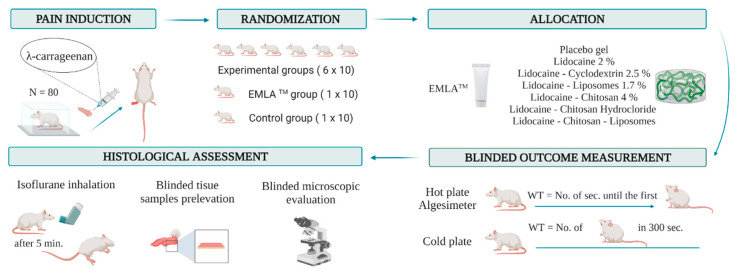
Research algorithm. Animal subjects were randomly allocated into eight groups after the carrageenan-induced inflammation. An experimental formula, placebo gel, or EMLA^TM^ cream was applied to the hind paw. Behavior tests were performed after 30, 60, 120, 180, and 240 min. For the hot plate and algesimeter, WT represents the time until the first sign of discomfort appears. For the cold plate, WT represents the number of movements secondary to the discomfort that occurred in the first 300 s. After the experiment, the animals were euthanized. Tissue samples were collected, and the side effects were histologically analyzed. WT: withdrawal threshold.

This randomized experimental study was performed and reported according to the ARRIVE 2009 Checklist [24], as indicated in Supplementary Materials. The research algorithm is schematically represented in Figure 1.

### 2.1. Formulation’s Preparation

**Figure 2:**
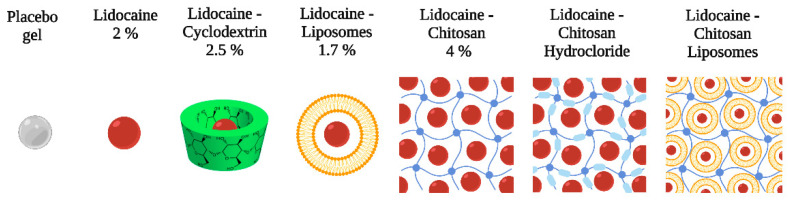
The substances applied in the experimental groups.

The substance used to induce hyperalgesia, λ-carrageenan, was diluted in fresh saline water (Sigma-Aldrich, Germany) and administered subcutaneously in order to produce inflammation. Placebo gel applied in the control group was a hydroxyethylcellulose-based commercial lubricant (Johnson & Johnson, New Brunswick, NJ, USA) purchased at a local pharmacy. In the EMLA^TM^ group, a cream containing 2.5% of each lidocaine/prilocaine was applied (AstraZeneca Ltd., London, UK). Lidocaine 2% gel by itself and in combination with another enhancing drug diffusion (cyclodextrin, chitosan, chitosan hydrochloride) was synthesized by “Petru Poni” Institute. Since the topical administration of analgesics remains a problem, several substances that can be used as carriers for the active substances have been described in the literature. Liposomes represent a variant allowing for a faster diffusion of the substance to be researched and a greater permeability and solubility, with minimal adverse effects and toxicity. The lidocaine-loaded liposomes were received from the “Gheorghe Asachi” Technical University of Iasi. The administrated dose of lidocaine was the same for each formulation. Liposomes were prepared from phosphatidylcholine and cholesterol by rehydration of a thin lipid film followed by extrusion. Liposomes act by penetrating the epidermis and then carrying the drug into the skin, where large multilamellar vesicles (MLV) could lose their external bilayer during penetration. Then, these liposome lipids penetrate into the stratum corneum by adhering onto the surface of the skin, subsequently destabilizing and fusing or mixing with the lipid matrix. Thereafter, they may act as penetration enhancers, loosening the lipid structure of the stratum corneum and promoting impaired barrier function of these layers to the drug, with less well-packed intercellular lipid structure forms, and with subsequent increased skin partitioning of the drug. The medium size of liposomes was around 112 nm, and the concentration used was 6.2 mg/mL (determined by Stewart assay). The substances applied in the experimental groups are schematically represented in Figure 2.

Small unilamellar vesicles (SUVs) were prepared by hydrating lipid films followed by ultrasound using a sonication probe (Bandelin Sonopuls, Berlin, Germany) for 1–2 cycles until the suspension became completely transparent. The SUV liposome suspension was then centrifuged at 15,000 rotations per minute (Hettich-Rotilabo, Karlsruhe, Germany) for 10–15 min in order to precipitate the titanium fragments resulting from detachment from the ultrasonic probe due to the high intensity used. Finally, the liposomes were incubated for 1–2 h at a temperature above the thermal transition temperature (25 °C for the use of phosphatidylcholine and 60 °C for 1,2-distearoyl-sn-glycerol 3-phosphocholine) to normalize structural defects in the liposomal membranes. The lidocaine–liposome formula was prepared by dispersing lidocaine in the aqueous phase of the liposomes, and then being stored at 4 °C before use.

The following materials were used to obtain SUV liposomes: phosphatidylcholine (LIPOID Gmbh, Ludwigshafen am Rhein, Germany), cholesterol (Sigma-Aldrich, St. Louis, MO, USA), chloroform (FLUKA—fully integrated Monte Carlo simulation package, with analytical purity greater than 99.5% and density 1.492 g/mL), methanol (Riedel de Haën, Charlotte, NC, USA, 99.5% purity), and a pH 7.4 phosphate buffer solution (prepared in the laboratory). Liposomes were characterized physicochemically by: (a) quantitative determination of phospholipids, (b) determination of liposome size, and (c) determination of LID from liposomes.

- Quantitative determination of phospholipids

**Figure 3:**
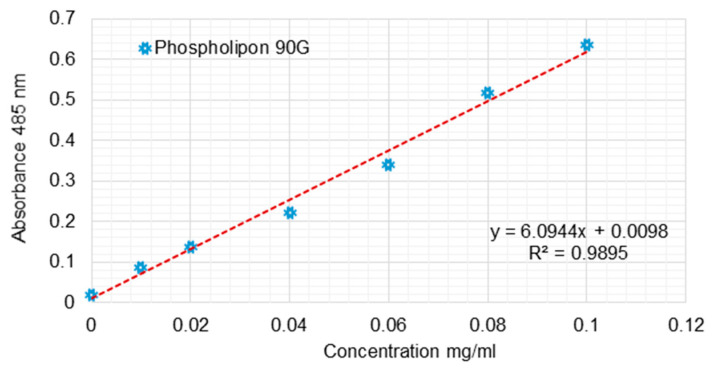
Calibration curve for the quantitative determination of the phospholipids using the Stewart method.

The Stewart method is a specific method used to identify phospholipids and utilizes the color reaction given by the complex formed between the phospholipid and the red ammonium ferrothiocyanate. It is soluble in chloroform in which it is extracted and measured by ultraviolet–visible spectroscopy. For these measurements, 1 L of Stewart reagent stock solution was prepared, sufficient for the whole set of experiments. The solution was made by dissolving 27 g of ferric chloride (FeCl_3_ x 6H_2_O) together with 30 g of ammonium thiocyanate (NH_4_SCN). The prepared solution was stable for several months, during which all the experiments lasted. In order to measure the amount of phospholipids in various samples, it was necessary to perform a calibration curve, given that the quantitative measurements were performed by ultraviolet–visible spectroscopy. After tracing the absorption spectrum of the solution formed by the complex between phospholipids and ammonium ferrothiocyanate, it was observed that the specific wavelength is 485 nm (Figure 3).

To determine the phospholipid concentrations of liposome solutions, a typical experiment consisted of placing in a test tube a 10 or 20 μL aqueous suspension of liposomes over which 2 mL of chloroform was added for the complete dissolution of lipids. In addition, two more milliliters of Stewart reagent was added, and the two phases were very well mechanically mixed to allow for the extraction of the entire amount of complex formed in the chloroform phase. The tube was then coated to prevent the chloroform’s evaporation from the bottom of the tube, and a complete separation of the two phases was made. After 15 min, the upper phase was removed with a Pasteur pipette, and then, using ultraviolet spectroscopy, the reading was made very quickly. The absorbed value of the measured sample was correlated with the corresponding value from the calibration curve. In this way, it was possible to determine the exact amount of phospholipids from the 10-20 μL suspension of liposomes and the 2 mL of chloroform in which their dissolution was performed. The concentration of the liposome suspension was then calculated mathematically. Thus, liposome suspensions with a total phospholipid content ranging from 4.4 to 6.2 mg/mL were obtained for SUV suspensions.

b. Determining the size of liposomes

**Figure 4:**
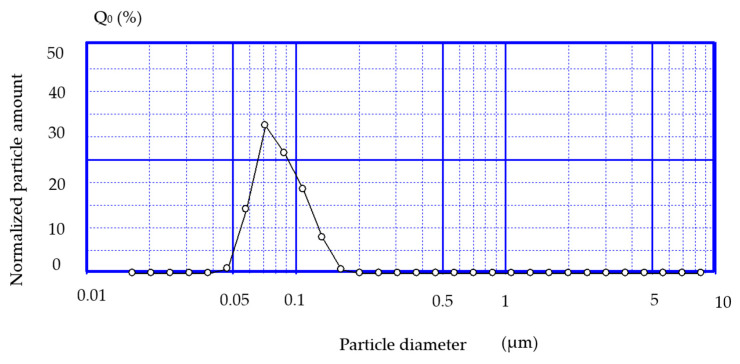
Dimensional distribution of lidocaine–cyclodextrin-loaded SUV liposomes.

The dimensional distribution for liposome dispersions was measured using dynamic light scattering using a SHIMADZU (Kyoto, Japan)—SALD 7001 instrument, which is capable of measuring the particle diameter distribution analyzed. One milliliter of liposomes suspension was introduced in twenty milliliters of PBS quartz cuvette, which was equipped with a slow stirring device to maintain the homogeneity of the suspension. The concentration of the liposomes was adjusted, diluting/concentrating the suspension until the intensity of the signal was 50% for all analyzed formulations. Previously, the blank for PBS was made. Thus, it was observed that the size of the SUV liposomes is relatively uniform, the resulting average diameter being 86 nm (Figure 4).

c. Determination of lidocaine in liposomes

**Figure 5:**
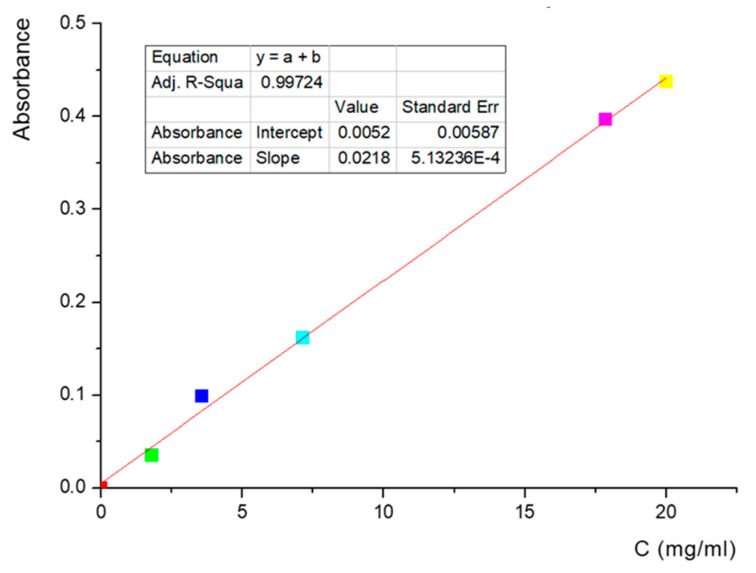
Lidocaine–cyclodextrin calibration curve in water—263 nm wavelength.

A liposome suspension was prepared to obtain liposomes loaded with lidocaine. A total of 2 mL of lidocaine solution (18 mg/mL) was used to hydrate the lipid film. To determine the amount of lidocaine included in the liposomes, we used the indirect method of estimation for the liposome suspension that was centrifuged, and the supernatant was separated and analyzed spectrophotometrically. The determination of lidocaine in liposomes was performed by breaking the liposome vesicles with a surfactant (TRITON 100, Midland, MI, USA), and the spectrophotometric determination of the lidocaine–cyclodextrin complex was carried out by reporting the values obtained to the calibration curve shown in Figure 5. The obtained value has been compared with the value obtained after the spectrophotometrical determination of non-entrapped lidocaine. For analyzing the lidocaine loaded in liposomes for the calibration curve and for the blank PBS, TRITON was used (TRITON 100 presents a peak at 280 nm, and we checked that this does not interfere with LID complex). 

Two parameters were analyzed for the amount of LID complex entrapped in the liposomes to be determined: encapsulation efficiency (EE%) (Equation (1)) and the drug/lipid ratio between LID complex and the phospholipid content of the liposomes (Equation (2)).

(1) $$\mathrm{EE}\;\left(\%\right)=\frac{\mathrm{Encapsulated}\;\mathrm{LD}\;\left(\mathrm{mg}\right)}{\mathrm{Total}\;\mathrm{LD}\;\left(\mathrm{mg}\right)}$$

(2) $$\mathrm{DL}\;\left(\%\right)=\frac{\mathrm{Encapsulated}\;\mathrm{LD}\;\left(\mathrm{mg}\right)}{\mathrm{Total}\;\mathrm{PC}\;\left(\mathrm{mg}\right)}$$

The exact amount included in the liposomes was determined by making the difference between the initial value and the actual one, namely 9.7 mg lidocaine.

The encapsulation efficiency of the tested formulation was calculated taking into account 9.7 mg entrapped and the 36 mg of LID complex from 2 mL solution used for phospholipid film hydration, resulting in an efficiency of encapsulation of 27%, which we consider a good value due to the fact that, during extrusion, part of the drug is lost. Also, the drug/lipid ratio was calculated considering 9.7 mg LID complex and 12.4 mg of phospholipid content at every mL liposomal suspension. In conclusion, the drug/lipid ratio is 0.78 mg complex at every mg of phospholipid. 

Cyclodextrin used for the complexation of lidocaine is an oligolictide-modified compound with an average molecular weight of 1700 Da. β-cyclodextrin (Cyclolab, Budapest, Hungary) was dried with an Abderhalden’s drying pistol with P_2_O_5_ under vacuum at 80 °C for 72 h and kept in the desiccator over P_2_O_5_ under Ar atmosphere. Dextro, levo-lactide (Purac, AC Gorinchem, The Netherlands) was recrystallized from toluene, sublimated, and kept in the desiccator under an Ar atmosphere. The synthesis was performed by solution (anhydrous dimethylformamide, Sigma-Aldrich St. Louis, MO, USA) ring-opening polymerization of dextro, levo-lactide in the presence of β-cyclodextrin [25]. Cyclodextrin–lidocaine complexes were prepared by ethanol coprecipitation. The Stewart method was used for the quantitative determination of phospholipids, and liposomal suspensions were obtained for cyclodextrin–lidocaine SUV with a molar concentration of phosphatidylcholine/cholesterol/lidocaine of 6.5/3.25/1. MRI analysis confirmed the formation of lidocaine inclusion complexes in the cyclodextrins modified with lactide cavity, and the complex stoichiometry was 1:1.

The lidocaine transdermal delivery was measured in preliminary studies of preclinical analysis. In this stage, we optimized the preparation process and established the reaction conditions and sample selection. We used synthetic membranes and skin from the experimental animals. Lidocaine release was achieved using 2% chitosan solutions containing 4% lidocaine, first through a cellulose acetate membrane for dialysis and then through laboratory-excised rat skin after chemical hair removal. The experimental system used a Franz-type diffusion cell. Samples of 0.5 mL were collected at predetermined time intervals and analyzed by UV spectroscopy at 263 nm to determine the lidocaine content. The results confirmed Higuchi-type release kinetics with a maximum release threshold of ~90%.

### 2.2. Experimental Animals

In our study, we included a total number of 80 male adult rats, thus providing the balance between the ethical aspects and the statistical power of the group. All experimental animals were purchased from the “Victor Babeş” National Institute for Research and Development in Pathology and Biomedical Sciences, Bucharest, Romania. Inclusion criteria: Wistar race, male, minimum age three months and maximum four months, and weight between 180 g and 200 g. Exclusion criteria: unhealthy animals (expressed by the refusal of food or oral hydration), the presence of pathological changes in skin and hair (e.g., alopecia), and any other abnormalities. All the rats were included in the analysis. The rats were kept in a room with 12 h of light/12 h of dark circadian rhythm and had a controlled temperature (21 ± 2 °C). Before applying the procedures in the experiment, they were allowed to accommodate to these conditions for at least 24 h. In each cage stayed only one rat that had unlimited food and water. The experiment used polycarbonate rat cages that were standardized at 1500 cm^2^ volume.

### 2.3. Ethical Approval

The study was performed after obtaining approval from the Ethics Committee of the “Grigore T. Popa” University of Medicine and Pharmacy, Iaşi, Romania. This experimental study was carried out in accordance with the recommendations of the European Community regarding the use of drugs in preclinical studies, in conformity with the conventions of good laboratory practice, published in the Official Gazette, Part I No. 102 from 6 February 2002 (approved by the Government of Romania, Decision No. 63 from 24 January 2002), as well as in accordance with clinical norms and protocols regarding the testing of drugs (approved by the Ministry of Public Health No. 906 from 25 July 2006). In addition, nociceptive tests were performed in accordance with the guidelines reported in EU Directive 2010/63/EU [26], “Guiding Principles in the Care and Use of Animals” approved by the American Physiological Society and “Ethical Guidelines for Investigations of Experimental Pain in Conscious Animals”.

### 2.4. Carrageenan-Induced Inflammation

**Figure 6:**
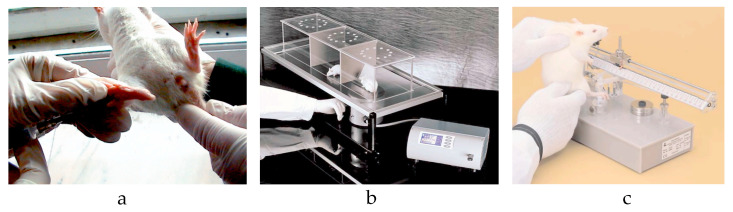
(**a**) λ-carrageenan injection. (**b**) Hot plate test. (**c**) Algesimeter test.

To assess the effect of the novel topical analgesic on inflammatory pain, we subcutaneously administered 10 µL of 1% λ-carrageenan in the ventral side of the right hind paw of the rats (Figure 6a). Before injection, the animal’s hair was removed with rodent’s depilatory cream. After administration, the animals were immediately placed in acrylic boxes for observation, and the local inflammatory status was monitored periodically. Inflammation was considered as at its peak approximately two hours and forty minutes after injection.

### 2.5. Group Allocation and Blindness

In order to evaluate the analgesic effects of the lidocaine-based formulation tested, the rats were randomly divided into eight groups (n = 10 in each group). The random.org platform was used for randomization. Once the λ-carrageenan-edema of the paw was obtained, the rats were divided according to the topical substance applied on the right hind paw:

- Group C (control, hydroxyethylcellulose-based lubricant);
- Group E (EMLA^TM^);
- Group L (lidocaine 2%);
- Group L-CD (lidocaine + cyclodextrin 2.5%);
- Group L-LP (lidocaine + liposomes 1.7%);
- Group L-CS (lidocaine + chitosan 4%);
- Group L-CSh (lidocaine + chitosan hydrochloride);
- Group L-CS-LP (lidocaine + chitosan + liposomes).

All the substances tested together with the placebo had the same pharmaceutical form (gel). In contrast, EMLA^TM^ had a cream texture; thus, a full allocation concealment could not be realized. An investigator that was not involved in the experiment prepared the substances with cream or gel texture and introduced them in pharmaceutical tubes (10 g each). The recipients were entirely covered with opaque tapes and were labeled with sequential numbers according to the randomization. The recipients were delivered to a researcher who participated only in this experiment stage and was blinded to the group assignment. Regarding the dose, a thin layer of cream or gel was applied for each substance. Two experienced researchers measured the outcome. While one operated the behavioral test and evaluated the WT, the other collected tissue samples. The histological assessment was conducted by a pathologist. All of them were blinded to group allocation during the entire study period.

### 2.6. Behavioral Test

The rats were tested to determine their WT to thermal stimuli using a hot plate (HP) and cold plate (CP). The discomfort was defined by the appearance of any behavior change: hind paw licking, forepaw licking, hindleg withdrawal, leaning posture, jumping off, stamping, and freezing, followed or not by immobile sniff and walk sniff. For HP, rats were individually placed on a plate maintained at 55 °C ± 0.1 °C (Ugo Basile Hot Plate, DS 37, Gemonio, Italy) [27]. The time until the first sign of discomfort determined the WT. To prevent tissue damage, the cut-off point was set to 12 s (Figure 6b). For CP, the animals were placed on a plate thermostatically maintained at 5 °C (Ugo Basile Cold Plate, 35100, Italy) [28]. The total number of movements secondary to the discomfort that occurred in the first five minutes determined the WT. The evaluation of WT to mechanical stimuli was performed using an algesimeter (Ugo Basile Analgesy-Meter, 7200, Italy) [29]. After the rat’s paw was placed on a plane, a pressure with progressive force (16 g/second) was exerted on it. The time until the paw retracts determined the WT. The cut-off point was set to 20 s (Figure 6c). In all groups, the WT was assessed at baseline and at specified intervals after the topical application: 30, 60, 120, 180, and 240 min.

### 2.7. Euthanasia of the Experimental Animals

The adult Wistar rats were euthanized at 4.5 h after topical application, without any mental or physical suffering. This procedure was performed in a separate room designed just for the necropsy, being thus separated from the room where the animals were kept daily. In just a couple of seconds, the inhalation of an anesthetic (isoflurane) induces unconsciousness. After about 5 min later, we observed the absence of vital signs. After the death had been confirmed, tissue samples were collected.

### 2.8. Histopathological Assessment

For this, tissue was collected from the hind paw involved in the experiment. The sample collection was performed by paraffin inclusion and microtome sectioning at 3–5 μm. The samples were stained with both eosin-hematoxylin and a Goldner–Szekely trichrome stain. All samples were observed microscopically for any histopathological change.

### 2.9. Statistical Analysis

All statistical analyses were carried out using SPSS v 20.0 (SPSS Inc., Chicago, IL, USA) and Excel software. In our study, we used independent variables represented by the time in which the substance was applied, and dependent variables that included the results after each substance application. We compared the substances and their potency at 30 min, 60 min, 120 min, 180 min, and 240 min. Given the continuous variables used, paired method of data collection, and paired t-test used as statistic test, we applied the appropriate sample size formulation: sample size = (Z1-α/2 + Z1-β)2 SD2/d2, where Z1-α/2 = Z 0.05/2 = Z 0.025 = 1.96 (from Z table) at type 1 error of 5% and Z1-β = Z0.20 = 0.842 (from Z table) at 80% power [30]. We borrowed SD from the pilot study, which investigated the gel formulas containing lidocaine and liposomes [31]. Based on previous studies, we estimated that the minimum difference between mean values will be 2.5 to be considered clinically significant, representing the effect size used in the formula as d. We applied the normality tests represented by Shapiro–Wilk and Kolmogorov–Smirnov tests on our results for each used substance. They had a significant value above 0.05, thus accepting the null hypothesis and presuming the fact that our parameters of the substances were normally distributed. The values were similar both in Shapiro–Wilk and Kolmogorov–Smirnov tests. Data were expressed as means, medians with interquartile range, or percentages, as appropriate. Repeated measures analysis of variance (ANOVA) was used to assess time and substance effect. The significance level was set a priori at *p* < 0.05.

## 3. Results

### 3.1. Primary Outcomes

The anesthetic effect of the experimental pharmaceutical formulation was determined by the inclusion or not of a drug enhancer. If a carrier was used, the groups’ differences were influenced by the type of behavioral test used and by the time they were performed. The WT value assessed the behavioral response specific to the pain test. The mean and 95% confidence interval for all the experimental substances, along with the p-value resulting from the comparison with the control and EMLA group, are reported in Tables S1–S6 from Supplementary Materials. Statistical indicators for the researched substances, such as minimum, maximum, and standard deviation values, are found in Tables S7 and S8 from Supplementary Materials. We have summarized in Table 1 the results obtained for the different substances that we used.

#### 3.1.1. Hot Plate

**Figure 7:**
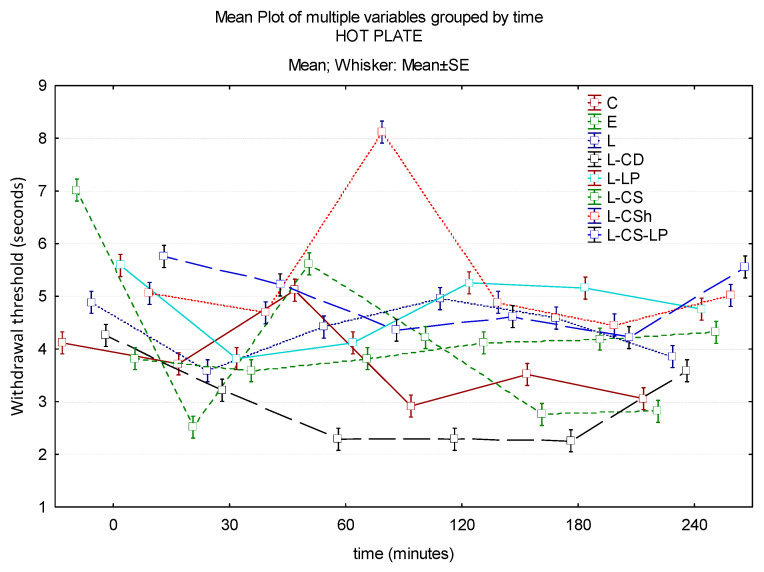
Time–effect curves of the topical substances on the hot plate. WT is plotted versus time. The horizontal axis expresses the time from the administration of the gels on the hind paw to the moment of the behavior test. The vertical axis expresses the time to the first sign of discomfort indicated by WT. The analgesic activity of the formulas is directly proportional to the number of seconds of the WT; WT: withdrawal threshold, C: control, E: EMLA^TM^, L: lidocaine 2%, L-CD: lidocaine + cyclodextrin 2.5%, L-LP: lidocaine + liposome 1.7%; L-CS: lidocaine + chitosan 4%, L-CSh: lidocaine + chitosan hydrochloride, L-CS-LP: lidocaine + chitosan + liposome.

At 30 min after the application of each of the substances, their analgesic effect is superior to EMLAs. L-CS-LP has the strongest effect, and L-CD has the weakest. Regarding the potency, a statistically significant difference is found between the effect obtained by EMLA and L-CS (*p* ≤ 0.001). At 60 min, the maximum analgesic effect is recorded by L-CSh, followed by EMLA. A lower analgesic effect compared to EMLA is found when using L, L-CS-LP, L-LP, L-CS, and L-CD. From this last category, L-CS-LP has the best effect, a statistically significant lower effect compared to EMLA (*p* = 0.018). At 180 min, the maximum analgesic effect is in L-LP, followed by L. The weakest effect is observed in L-CD and EMLA, between L and the control group, it being a statistically significant difference (*p* = 0.027). In conclusion, the action of L-CS-LP was superior to EMLA, except for the determination at 60 min, the possible late effect being due to the time required for the gradual release of the active substance from the liposomes. The long-term effect is later, but long-lasting. The L-CSh effect is superior to EMLA throughout the experiment and remains approximately constant. Of all of the tested substances, L-CD had the minimum analgesic effect during the entire experiment. Time–effect curves of the topical substances on the hot plate are represented in Figure 7.

#### 3.1.2. Cold Plate

**Figure 8:**
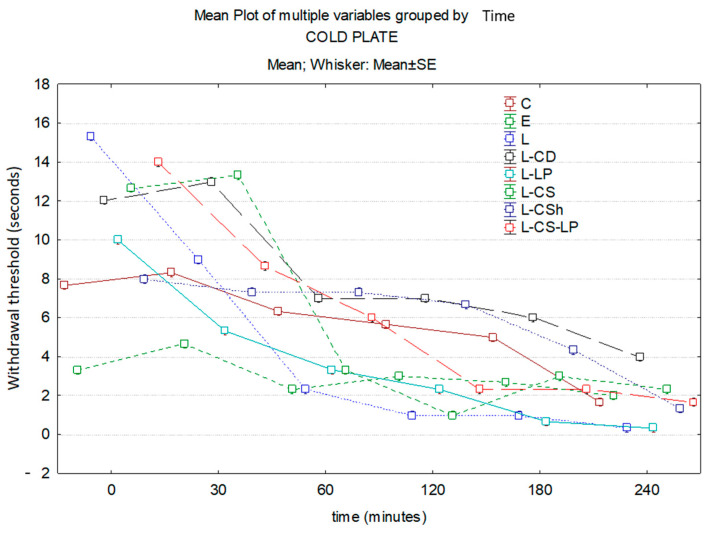
Time–effect curves of the topical substances on the cold plate. WT is plotted versus time. The horizontal axis expresses the time from the administration of the gels on the hind paw to the moment of the behavior test. The vertical axis expresses the total number of movements secondary to the discomfort that occurred in the first five minutes indicated by WT. The analgesic activity of the formulas is inversely proportional to the number of seconds of the WT; WT—withdrawal threshold, C: control, E: EMLA^TM^, L: lidocaine 2%, L-CD: lidocaine + cyclodextrin 2.5%, L-LP: lidocaine + liposome 1.7%; L-CS: lidocaine + chitosan 4%, L-CSh: lidocaine + chitosan hydrochloride, L-CS-LP: lidocaine + chitosan + liposome.

At 30 min, EMLA has the highest effect, followed by L-LP. At this time, there is a statistically significant difference between L-LP and control (*p* ≤ 0.001). This statistically significant difference is maintained at both 60 and 120 min (all *p* ≤ 0.001). The analgesic effect is maintained until the end of the experiment, but without statistical significance. At 120 min, the maximum analgesic effect is observed in L-CS. On the other hand, EMLA has a superior analgesic effect compared to L-CSh with a statistically significant difference (*p* = 0.043). At 180 min, the effect of L-CS becomes inferior to EMLA, it being maintained until the end of the experiment. At the end of the experiment, L has a maximum effect compared to the control group, with a statistically significant difference (*p* ≤ 0.001) between the values obtained. The substance with the lowest analgesic effect at 120 min is L-CD, it being maintained until the end of the experiment. Time–effect curves of the topical substances on the cold plate are represented in Figure 8.

#### 3.1.3. Algesimeter

**Figure 9:**
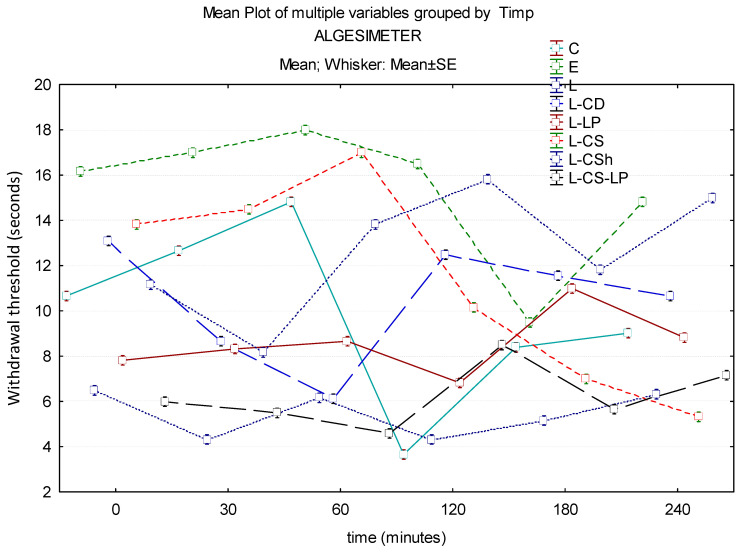
Time–effect curves of the topical substances on algesimeter. WT is plotted versus time. The horizontal axis expresses the time from the administration of the gels on the hind paw to the moment of the behavior test. The vertical axis expresses the time to the first sign of discomfort indicated by WT. The analgesic activity of the formulas is directly proportional to the number of seconds of the WT; WT—withdrawal threshold, C: control, E: EMLA^TM^, L: lidocaine 2%, L-CD: lidocaine + cyclodextrin 2.5%, L-LP: lidocaine + liposome 1.7%; L-CS: lidocaine + chitosan 4%, L-CSh: lidocaine + chitosan hydrochloride, L-CS-LP: lidocaine + chitosan + liposome.

In the case of pain testing with the help of the algesimeter, the maximum effect of the substances to be researched is represented by L-CS. Group L has a low effect throughout the experiment, with statistically significantly lower results compared to EMLA at 30 min (*p* ≤ 0.001) and 240 min (*p* = 0.027). At 60 min, the maximum effect is observed in EMLA and the minimal effect is observed in L-CS-LP, with a borderline statistical difference (*p* = 0.054) between the two. Another substance with low potency is L-LP, which at 30 min has a net lower effect than control (statistically significant, *p* ≤ 0.001). At the end of the experiment, the L-CS effect is minimal, it being exceeded by L, L-CS-LP, L-LP, L-CD, and L-CSh. There is a statistically significant difference between EMLA and L-LP (*p* = 0.049). The time–effects curves of the topical substances on the algesimeter are represented in Figure 9.

In order to objectify the homogeneity of the studied group, we considered it necessary to quantify the minimum, maximum, and standard deviation. These parameters allow us to have a better comparison of the used substances at different time intervals, values represented in Tables S7 and S8 from Supplementary Materials.

**Figure 10:**
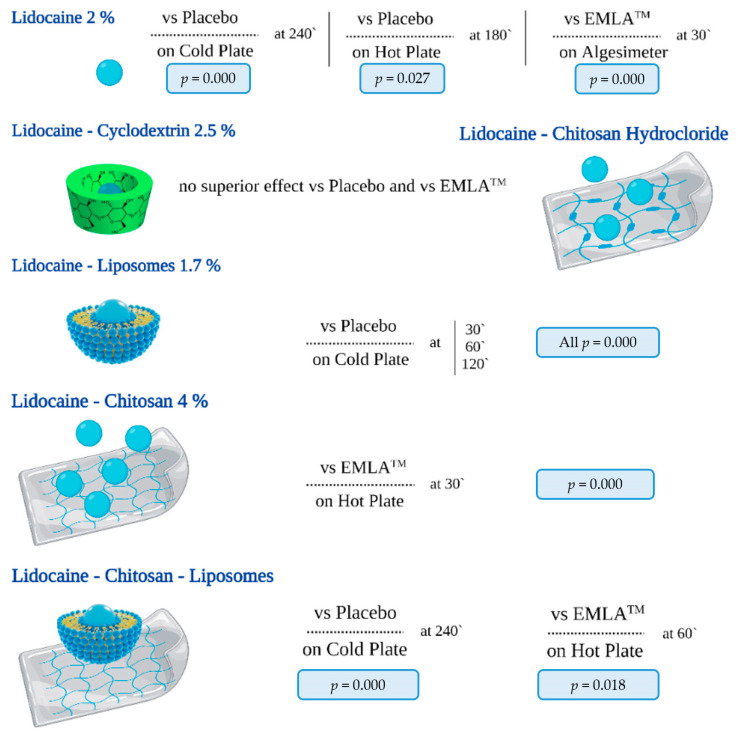
The effects of the tested formulations compared to placebo and EMLA^TM^. Three of the studied drug enhancers had statistically significant benefits in combination with lidocaine–liposomes, chitosan, and chitosan liposomes. Of these, only one formula showed significantly greater benefits compared to placebo at three moments of the pain assessment (at 30, 60, and 120 min) from carrageenan injection lidocaine–liposomes 1.7%.

Therefore, cyclodextrin was the drug carrier with the lowest favorable results, without achieving an effect size with a significant difference compared to placebo and EMLA^TM^. Compared to EMLA, the use of chitosan hydrochloride as a matrix of hydrogel for lidocaine did not demonstrate any benefits in the behavioral assessment with the cold plate. In contrast, using chitosan 4% had a more important effect, proven by the hot plate test. Although these studied enhancers offer important hopes for the future, lidocaine 2% had beneficial effects both compared to placebo in the cold and hot plate and with EMLA^TM^ in the algesimeter. Thus, the increase in the withdrawal threshold in the present study was related to both the analgesic and antihyperalgesic effects of lidocaine. The superior effects of the tested formulas compared to placebo and EMLA^TM^ are illustrated in Figure 10.

### 3.2. Secondary Outcomes

#### 3.2.1. Macroscopic Evaluation

**Figure 11:**
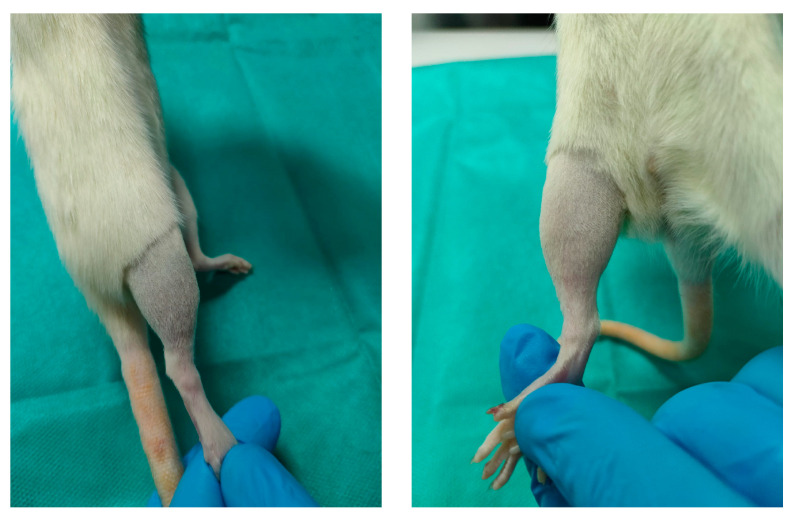
Macroscopic evaluation.

Looking after the side effects at the administration site was another endpoint of the study. From a macroscopic point of view, no skin discoloration or skin inflammation occurred throughout the experiment (Figure 11).

#### 3.2.2. Microscopic Evaluation

**Figure 12:**
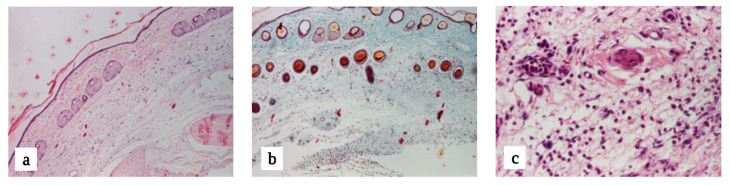
(**a**) Control group: hematoxylin–eosin × 40, normal skin. (**b**) EMLA group: Goldner–Szekely trichrome × 40. (**c**) Experimental Group C: hematoxylin–eosin × 200.

In both hematoxylin–eosin and Goldner–Szekely trichrome staining, skin samples collected from the control animals had a normal architecture. The pilosebaceous, apocrine, and eccrine glands were free of pathological changes. Skin samples from the EMLA and experimental groups indicated acute inflammatory reactions: polymorphonuclear neutrophils, diffuse disposition in the superficial and deep dermis, particularly in the deep dermis, perivascular disposition, edema, and vascular congestion with leukocytosis. Specific for the experimental groups, perineural dispersion and focal areas of microhemorrhages were identified. All groups were free of side effects, including local necrosis, allergic reactions, arteriolar spasms, and direct nerve toxicity (Figure 12).

## 4. Discussion

### 4.1. Summary of Evidence

Currently, the only worldwide available topical anesthetic for intact skin is EMLA^TM^ (Eutectic Mixture of Local Anesthetics; AstraZeneca Ltd., London, UK). Currently, the pharmaceutical formulation on the market is a 5% cream preparation, containing equal quantities of lidocaine and prilocaine [32]. Although an increase in skin blood flow and vascular reactivity was initially reported when using EMLA [33], recent studies demonstrate that applying EMLA cream for 60 min has no effect on skin blood flow [34]. Regarding the anesthetic effect, the nerve impulse conducted through the afferent cutaneous sensory nerve is attenuated. Other pathways may also exist, but it is essential to mention EMLA’s inhibition of the sympathetic cholinergic active vasodilator nerve fibers [35]. Our results are in concordance with the available literature indicating that EMLA© topical administration is only slightly effective in reducing tactile sensitivity in newborn rats [36]. Another clinical trial showed that EMLA© partially decreases pain during tympanocentesis in less than a third of the patients [37].

Topical lidocaine is widely used in current practice for a variety of pain conditions. Lidocaine, presented as a gel pharmaceutical form, is an analgesic option for several vulnerable patients [38,39]. Once absorbed, lidocaine binds predominantly to alpha-1 glycoprotein acid and passively diffuses through the blood–brain barrier and the placenta. The liver metabolizes it into inactive metabolites that are subsequently renally excreted. No dose adjustment for liver and kidney function is required for this administration of lidocaine as a gel [40]. Topical lidocaine has been approved in several countries by the health authorities for the treatment of post-herpetic neuralgia [41]. There is also clinical evidence that lidocaine delivered through a transdermal system has diminished peripheral neuropathic pain associated with diabetic peripheral neuropathy, postsurgical or post-traumatic pain, and scar pain [42]. Other indications suggested by some authors, but requiring further studies, are carpal tunnel syndrome [43], knee osteoarthritis [44], and chronic low back pain with a neuropathic component [45]. Several hydrogel substances containing lidocaine have proven beneficial in local dentistry anesthesia [46].

In the present study, the best anesthetic effects had lidocaine incorporated into liposomes (lidocaine-in-liposomes) proven on the cold plate test compared to placebo. By mixing lidocaine-in-liposomes with a chitosan hydrogel (lidocaine-in-liposomes-in-chitosan), the formula had a significantly greater effect on cold plate evaluation compared to placebo and on hot plate evaluation compared to EMLA^TM^. For lidocaine-in-liposomes, the encouraging results are mainly due to an improved adhesiveness, retention time, and slow-release rate, thus prolonging the anesthetic effect of this water-soluble molecule. Regarding the favorable results that we obtained for lidocaine-in-liposomes-in-hydrogel, we consider that they are due to increased adhesion and gradually decreased water vapor. This mechanism was recently demonstrated by Peers et al., them being the first investigators to follow these systems with environmental scanning electron microscopy, using observation conditions as close as possible to a native state. In addition, the authors showed that the presence of lidocaine-in-liposomes inside the polymer matrix of hydrogel does not alter the rheological properties [47]. 

The secondary objective of our study was to determine the side effects of the substances at the level of their application. Throughout the experiment, no skin discoloration or skin inflammation occurred. At the end of the experiment, microscopic evaluation of skin samples showed an inflammatory reaction following λ-carrageenan injection. The epidermis, together with the superficial and deep dermis, have kept their normal architecture. All groups were free of side effects, including local necrosis, allergic reaction, arteriolar spasm, and direct nerve toxicity. Consistent with the results of our study, Wang et al. have shown that lidocaine encapsulated in liposomes loaded with transactivation transcriptional activator peptides achieves a stronger therapeutic effect along with no local side effects [48]. By mixing lidocaine incorporated into liposomes with a chitosan hydrogel, Li et al. have shown that this mixture not only prolongs the duration of action but also decreases the side effects of local anesthetic agents [49]. Regarding chitosan used as a carrier for lidocaine, Anirudhan et al. showed that, by adding hyaluronic acid, the rats were free of skin inflammation [50]. No studies that evaluated the effects of lidocaine incorporated into cyclodextrin have described the side effects of the substance in vitro or in vivo.

Kristl et al. studied the drug carriers, and important attention was accorded to chitosan. They described the safety usage of this natural polymer, helping in the regulation of the drug release. The chitosan that has a high molecular mass is rather used for constant release substances, moderately hydrolyzed for a better drug solubility [51]. In addition, Taveira et al. reported that chitosan was capable of a better drug dispersion in the profound layers of the skin due to its positive charge that interacts with the negative one from the skin [52]. In the literature, several studies have looked at the usefulness of chitosan specifically for transdermal lidocaine delivery. Liu et al. have encouraging results on lidocaine loaded with chitosan and zinc ferrite (ZnFe_2_O_4_) nanoparticles. The anesthetic efficiency of this formulation was improved by a high biocompatibility and sustained drug release in the physiological pH environment (4.8, 5.5, and 7.4) and temperature-responsiveness (25 and 37 °C) of normal tissues [53]. Anirudhan et al. have proven benefits for a novel transdermal device containing a hyaluronic acid, lidocaine, and chitosan matrix, improving both the skin release of lidocaine and adhesion [50]. According to Zhang, chitosan and hyaluronic-acid-modified lipid nanoparticles are involved in a layer-by-layer technique with encouraging results for local lidocaine administration [54].

Another drug delivery system used for the topical administration of lidocaine is liposomes, described for the first time by Mezei et al. [55]. The time required to install analgesia should be reduced as much as possible, and this can be performed by local administration of the anesthetic. To achieve this goal, liposomes that include anesthetics can be used in the treatment of pain. Liposomes allow for a firmer adhesion to the skin by creating a film on the contact with the dermis, and then gradually releasing the active substance, without losing water from the transepidermal tissue [54,56]. A recent study demonstrated that transdermal delivery using elastic nano-liposomes and microneedle array pretreatment enhanced the delivery of lidocaine hydrochloride on mice’s skin. In addition, this transdermal delivery prolonged the anesthetic effect in vivo compared to the one observed by lidocaine hydrochloride application alone. In vitro, no group showed significant cytotoxicity on the human immortalized keratinocyte cell line HaCaT up to an elastic nano-liposomes concentration of 100 µg/m [57].

Since 1997, some researchers have considered using both chitosan and liposomes, this causing an increase in the stability of the product with a prolonged release of the active substance [58] that can cross the mucosal barrier [59] and has an increased permeability to the skin [60]. Hurler et al. developed a biofilm for wound dressings, a novel drug delivery system for mupirocin (mupirocin-in-liposomes-in-chitosan). The investigators used the in vivo mice burn model, showing that the system is equally good and safe for administration onto the wounded site. They obtained a faster release of mupirocin from liposomes incorporated into hydrogels [61]. Despite these discouraging results, studies that included lidocaine as a delivery drug for these complex systems have shown the opposite. Li et al. demonstrated that formulations based on chitosan and liposomes are better penetration enhancers for lidocaine compared with lidocaine-loaded chitosan [49]. In the last few years, the interest in this formula has increased considerably. Peers et al. succeed in embedding liposomes into chitosan physical hydrogel for the delayed release of lidocaine without implying the modification of their rheological properties [47].

Cyclodextrin is another active substance transporter solution for lidocaine due to its properties of including the active substance in complexes. Thus, lidocaine becomes more soluble and improves its physical and chemical properties, as well as its release. The newly formed product results in a slower release of inflammation mediators at the site of administration, which causes an increase in absorption and permeability at the cellular level. In addition, cyclodextrins are expected to increase safety and increase the permeability and release of the active substance in the skin [62]. Cyclodextrin used as a drug delivery vehicle has improved the efficacy and safety profile for several molecules, with predominant bioapplicability in oncology, including doxorubicin [63], paclitaxel [64], and interleukin 12 [65]. Another area in which cyclodextrin is of great interest is the one of infectious diseases. Ho et al. showed an improvement in the solubility and release of the ciprofloxacin [66], while Haley et al. have shown a reduction in adverse effects and an improvement in the antifungal effect of amphotericin B [67].

### 4.2. Limitations

Several limitations of our study need to be considered in future research. First, given the heterogeneity of the pharmaceutical consistency, complete concealment of the allocation was impossible. However, we aimed to reduce this source of bias by grouping the formulas into two types of distribution: cream tube and gel tube. Secondly, we did not evaluate the long-term effects of lidocaine with or without a nanocarrier on pain, as our study period between inflammation induction and euthanasia was only 4.5 h. In addition, blood level and systemic side effects were not assessed. Third, isoflurane used for general anesthesia has the potential to affect the inflammatory reaction. Given the short exposure time to isoflurane and the low inhaled concentration, this would not significantly affect our results. In reducing the confounding variable role, isoflurane was applied to rats in all groups. Given the acute pain model reproduced in the present study by λ-carrageenan injection, these results cannot be generalized to chronic pain. Finally, the Wistar rat model in our study cannot represent all cases of acute pain. To enhance the possibility of clinical application, further research with other animal models is needed.

## 5. Conclusions

The appearance of the investigated carriers that improve the penetration capacity of the substances allows for the installation of analgesia in a much shorter time on one hand, and, on the other hand, they can be easily used without systemic side effects. The best analgesic effect was observed with the L-LP compound for the cold plate at evaluations of 30, 60, and 120 min. This finding paves the way for new research on the ability of liposomes to penetrate the surface layers of the skin and allow for a gradual dispersion of the active substance. Once chitosan is added, it does not allow lidocaine to be released as quickly from the liposomes, but it has a late effect, as seen at the end of the experiment (240 min). It is appreciated that the investigated formulations do not cause local lesions. Several studies are further needed to confirm the role of liposomes as a carrier for transdermal delivery systems and to determine the maximum dose of the active substance that causes the maximum analgesic effect but with the minimal adverse effect.

## Figures and Tables

**Table 1:** The results obtained after applying the used substances.

	Control	EMLA	Lidocaine 2%	Lidocaine–Cyclodextrin 2.5%	Lidocaine–Liposomes 1.7%	Lidocaine–Chitosan 4%	Lidocaine–Chitosan Hydrochloride	Lidocaine–Chitosan Liposomes
Hot Plate								
Baseline	4.13 s	7.03 s	4.9 s	4.27 s	5.6 s	3.83 s	5.07 s	5.77 s
30′	3.73 s	2.53 s	3.6 s	3.23 s	3.83 s	3.6 s	4.7 s	5.23 s
60′	5.13 s	5.63 s	4.43 s	2.3 s	4.13 s	3.83 s	8.13 s	4.37 s
120′	2.93 s	4.23 s	4.97 s	2.3 s	5.27 s	4.13 s	4.9 s	4.63 s
180′	3.53 s	2.77 s	4.6 s	2.27 s	5.17 s	4.2 s	4.47 s	4.23 s
240′	3.07 s	2.83 s	3.87 s	3.6 s	4.77 s	4.33 s	5.03 s	5.57 s
Cold Plate								
Baseline	7.67 s	3.33 s	15.33 s	12.03 s	10 s	12.67 s	8 s	14 s
30′	8.33 s	4.67 s	9 s	13 s	5.33 s	13.33 s	7.33 s	8.67 s
60′	6.33 s	2.33 s	2.33 s	7 s	3.33 s	3.33 s	7.33 s	6 s
120′	5.67 s	3 s	1 s	7 s	2.33 s	1 s	6.67 s	2.33 s
180′	5 s	2.67 s	1 s	6 s	0.67 s	3 s	4.33 s	2.33 s
240′	1.67 s	2 s	0.33 s	4 s	0.33 s	2.33 s	1.33 s	1.67 s
Algesimeter								
Baseline	10.67 s	16.17 s	6.5 s	13.1 s	7.83 s	13.83 s	11.17 s	6 s
30′	12.67 s	17 s	4.33 s	8.67 s	8.33 s	14.5 s	8.17 s	5.5 s
60′	14.83 s	18 s	6.17 s	6.13 s	8.67 s	17 s	13.83 s	4.6 s
120′	3.67 s	16.5 s	4.33 s	12.5 s	6.83 s	10.17 s	15.83 s	8.5 s
180′	8.4 s	9.5 s	5.17 s	11.57 s	11 s	7 s	11.83 s	5.67 s
240′	9.03 s	14.83 s	6.33 s	10.67 s	8.83 s	5.33 s	15 s	7.17 s

## Data Availability

Data are contained within the article and Supplementary Information. Additional data can be provided on request from the corresponding authors.

## Supplementary Materials

The following supporting information can be downloaded at: https://www.mdpi.com/article/10.3390/jcm13010271/s1. ARRIVE guidelines checklist; Table S1. Paw withdrawal threshold after lidocaine 2.5% administration; Table S2. Paw withdrawal threshold after lidocaine–cyclodextrin 2.5% administration; Table S3. Paw withdrawal threshold after lidocaine–chitosan 4% administration; Table S4. Paw withdrawal threshold after lidocaine–chitosan hydrochloride administration; Table S5. Paw withdrawal threshold after lidocaine–liposomes administration. Table S6. Paw withdrawal threshold after lidocaine–liposomes administration; Tables S7 and S8. Statistical indicators for the researched substances.

## Abbreviations

EMLA	Eutectic Mixture of Local Anesthetics
WT	Withdrawal threshold
C	Control
L	Lidocaine 2%
L-CD	Lidocaine–cyclodextrin 2.5%
L-LP	Lidocaine–liposome 1.7%
L-CS	Lidocaine–chitosan 4%
L-CSh	Lidocaine–chitosan hydrochloride
L-CS-LP	Lidocaine–chitosan–liposome
HP	Hot plate
CP	Cold plate
