# Supplementary material for: Lidocaine–Liposomes—A Promising Frontier for Transdermal Pain Management
Source: J Clin Med. 2024 Jan 3;13(1):271. doi: 10.3390/jcm13010271 (PMC10779996; doi:10.3390/jcm13010271)
Supplement: Supplementary file 1 [file jcm-13-00271-s001.zip › jcm-2748965-supplementary materials.pdf]

Table S1. Paw withdrawal threshold after lidocaine 2.5 % administration

	Time after λ-C delivery (minutes)	L (n=10)		Control group (n=10)		EMLA Group (n=10)		P Value	
		Mean	95% CI	Mean	95 % CI	Mean	95 % CI	L vs Control	L vs EMLA
**Cold plate**	0	15.333	(4.991-25.675)	7.666	(-2.675-18.009)	3.333	(1.899-4.767)	0.976	0.512
	30	9.000	(-7.289-25.289)	8.333	(3.162-13.504)	4.666	(-5.675-15.009)	0.761	0.403
	60	2.333	(-0.535-5.201)	6.333	(-0.837-13.504)	2.333	(-3.403-8.070)	0.667	0.667
	120	1.000	(-1.484-3.484)	5.666	(-2.318-13.652)	3.000	(-5.956-11.956)	0.901	0.821
	180	1.000	(-1.484-3.484)	5.000	(0.697-9.302)	2.666	(-4.922-10.255)	0.333	0.546
	240	0.333	(-1.100-1.767)	1.666	(-1.201-4.535)	2.000	(-0.484-4.484)	**0.000**	0.333
**Hot plate**	0	4.900	(4.242-5.557)	4.133	(1.477-6.789)	7.033	(4.377-9.689)	0.575	0.758
	30	3.600	(2.854-4.345)	3.733	(-1.371-8.838)	2.533	(2.389-2.676)	0.953	0.333
	60	4.433	(1.239-7.627)	5.133	(2.620-7.646)	5.633	(1.257-10.009)	0.536	0.748
	120	4.966	(2.219-7.714)	2.933	(0.625-5.241)	4.233	(0.496-7.970)	0.198	0.782
	180	4.600	(2.861-6.338)	3.533	(2.307-4.758)	2.766	(2.049-3.483)	**0.027**	0.758
	240	3.866	(2.498-5.234)	3.066	(2.779-3.353)	2.833	(2.116-3.550)	0.300	0.967
**Algesimeter**	0	6.500	(2.021-10.978)	10.666	(-0.874-22.207)	16.166	(2.88-29.447)	0.110	0.391
	30	4.333	(3.616-5.050)	12.666	(0.412-24.920)	17.000	(-0.210-34.210)	0.731	**0.000**
	60	6.166	(1.464-10.869)	14.833	(-7.707-37.373)	18.000	(-2.894-38.894)	0.645	0.716
	120	4.333	(2.436-6.230)	3.666	(-1.353-8.686)	16.500	(-1.542-34.542)	0.212	0.750
	180	5.166	(2.581-7.752)	8.400	(2.55-14.241)	9.500	(3.808-15.191)	0.869	0.367
	240	6.333	(-3.782-16.449)	9.033	(1.41-16.650)	14.833	(-7.707-37.373)	0.547	**0.027**

λ-C: λ-carrageenan; L: Lidocaine 2.5 %; EMLA: Eutectic Mixture of Local Anesthetics; CI: confidence interval

Table S2. Paw withdrawal threshold after lidocaine-cyclodextrin 2.5% administration

	Time after λ-C delivery (minutes)	L-CD (n=10)		Control group (n=10)		EMLA Group (n=10)		P Value	
		Mean	95% CI	Mean	95 % CI	Mean	95 % CI	L-CD vs Control	L-CD vs EMLA
**Cold plate**	0	12.033	(11.889-12.176)	7.666	(-2.675-18.009)	3.333	(1.899-4.767)	0.512	0.000
	30	13.000	(-9.079-35.079	8.333	(3.162-13.504)	4.666	(-5.675-15.009)	0.299	0.657
	60	7.000	(-10.389-24.389)	6.333	(-0.837-13.504)	2.333	(-3.403-8.070)	0.333	0.333
	120	7.000	(-2.936-16.936)	5.666	(-2.318-13.652)	3.000	(-5.956-11.956)	0.234	0.512
	180	6.000	(-4.828-16.828)	5.000	(0.697-9.302)	2.666	(-4.922-10.255)	0.073	0.285
	240	4.000	(-5.936-13.936)	1.666	(-1.201-4.535)	2.000	(-0.484-4.484)	1	0.667
**Hot plate**	0	4.266	(2.402-6.131)	4.133	(1.477-6.789)	7.033	(4.377-9.689)	0.946	0.763
	30	3.233	(0.846-5.620)	3.733	(-1.371-8.838)	2.533	(2.389-2.676)	0.476	0.237
	60	2.300	(1.554-3.045)	5.133	(2.620-7.646)	5.633	(1.257-10.009)	0.365	0.351
	120	2.300	(1.217-3.382)	2.933	(0.625-5.241)	4.233	(0.496-7.970)	0.662	0.358
	180	2.266	(1.749-2.783)	3.533	(2.307-4.758)	2.766	(2.049-3.483)	0.577	0.154
	240	3.600	(2.109-5.090)	3.066	(2.779-3.353)	2.833	(2.116-3.550)	0.333	1
**Algesimeter**	0	13.100	(-5.823-32.023)	10.666	(-0.874-22.207)	16.166	(2.88-29.447)	0.888	0.831
	30	8.666	(-0.488-17.822)	12.666	(0.412-24.920)	17.000	(-0.210-34.210)	0.862	0.130
	60	6.133	(3.138-9.128)	14.833	(-7.707-37.373)	18.000	(-2.894-38.894)	0.167	0.806
	120	12.500	(5.927-19.072)	3.666	(-1.353-8.686)	16.500	(-1.542-34.542)	0.121	0.917
	180	11.566	(9.735-13.397)	8.400	(2.55-14.241)	9.500	(3.808-15.191)	0.440	0.324
	240	10.666	(3.388-17.944)	9.033	(1.41-16.650)	14.833	(-7.707-37.373)	0.068	0.452

λ-C: λ-carrageenan; L-CD: lidocaine-cyclodextrin 2.5%; EMLA: Eutectic Mixture of Local Anesthetics; CI: confidence interval

Table S3. Paw withdrawal threshold after lidocaine – chitosan 4 % administration

	Time after λ-C delivery (minutes)	L-CS (n=10)		Control group (n=10)		EMLA Group (n=10)		P Value	
		Mean	95% CI	Mean	95 % CI	Mean	95 % CI	L-CS vs Control	L-CS vs EMLA
**Cold plate**	0	12.666	(-1.889-27.222)	7.666	(-2.675-18.009)	3.333	(1.899-4.767)	0.403	0.109
	30	13.333	(3.293-23.372)	8.333	(3.162-13.504)	4.666	(-5.675-15.009)	0.754	0.396
	60	3.333	(-1.837-8.504)	6.333	(-0.837-13.504)	2.333	(-3.403-8.070)	0.821	0.821
	120	1.000	(-1.484-3.484)	5.666	(-2.318-13.652)	3.000	(-5.956-11.956)	0.901	0.821
	180	3.000	(-3.572-9.572)	5.000	(0.697-9.302)	2.666	(-4.922-10.255)	0.121	0.091
	240	2.333	(-1.461-6.127)	1.666	(-1.201-4.535)	2.000	(-0.484-4.484)	0.454	0.121
**Hot plate**	0	3.833	(3.034-4.631)	4.133	(1.477-6.789)	7.033	(4.377-9.689)	0.537	0.355
	30	3.600	(3.169-4.030)	3.733	(-1.371-8.838)	2.533	(2.389-2.676)	0.713	**0.000**
	60	3.833	(3.316-4.350)	5.133	(2.620-7.646)	5.633	(1.257-10.009)	0.196	0.530
	120	4.133	(3.099-5.167)	2.933	(0.625-5.241)	4.233	(0.496-7.970)	0.890	0.130
	180	4.200	(1.219-7.181)	3.533	(2.307-4.758)	2.766	(2.049-3.483)	0.398	0.333
	240	4.333	(0.942-7.724)	3.066	(2.779-3.353)	2.833	(2.116-3.550)	0.428	0.905
**Algesimeter**	0	13.833	(-10.261-37.928)	10.666	(-0.874-22.207)	16.166	(2.88-29.447)	0.118	0.163
	30	14.500	(-1.357-30.357)	12.666	(0.412-24.920)	17.000	(-0.210-34.210)	0.399	0.870
	60	17.000	(-7.465-41.465)	14.833	(-7.707-37.373)	18.000	(-2.894-38.894)	0.936	0.297
	120	10.166	(-11.069-31.403)	3.666	(-1.353-8.686)	16.500	(-1.542-34.542)	0.611	0.351
	180	7.000	(5.757-8.242)	8.400	(2.55-14.241)	9.500	(3.808-15.191)	0.690	0.546
	240	5.333	(3.436-7.230)	9.033	(1.41-16.650)	14.833	(-7.707-37.373)	0.837	0.318

λ-C: λ-carrageenan; L-CS: lidocaine – chitosan 4 %; EMLA: Eutectic Mixture of Local Anesthetics; CI: confidence interval

Table S4. Paw withdrawal threshold after lidocaine - chitosan hydrochloride administration

	Time after λ-C delivery (minutes)	L-CSh (n=10)		Control group (n=10)		EMLA Group (n=10)		P Value	
		Mean	95% CI	Mean	95 % CI	Mean	95 % CI	L-CSh vs Control	L-CSh vs EMLA
**Cold plate**	0	8.000	(1.427-14.572)	7.666	(-2.675-18.009)	3.333	(1.899-4.767)	0.700	0.788
	30	7.333	(1.596-13.070)	8.333	(3.162-13.504)	4.666	(-5.675-15.009)	0.821	0.821
	60	7.333	(-8.829-23.496)	6.333	(-0.837-13.504)	2.333	(-3.403-8.070)	0.305	0.305
	120	6.666	(-5.072-18.406)	5.666	(-2.318-13.652)	3.000	(-5.956-11.956)	0.235	**0.043**
	180	4.333	(-10.222-18.889)	5.000	(0.697-9.302)	2.666	(-4.922-10.255)	0.109	0.103
	240	1.333	(-2.461-5.127)	1.666	(-1.201-4.535)	2.000	(-0.484-4.484)	0.879	0.788
**Hot plate**	0	5.066	(0.804-9.328)	4.133	(1.477-6.789)	7.033	(4.377-9.689)	0.277	0.460
	30	4.700	(2.961-6.438)	3.733	(-1.371-8.838)	2.533	(2.389-2.676)	0.622	0.091
	60	8.133	(-4.308-20.574)	5.133	(2.620-7.646)	5.633	(1.257-10.009)	0.570	0.146
	120	4.900	(0.299-9.500)	2.933	(0.625-5.241)	4.233	(0.496-7.970)	0.235	0.745
	180	4.466	(3.462-5.470)	3.533	(2.307-4.758)	2.766	(2.049-3.483)	0.177	0.909
	240	5.033	(1.134-8.932)	3.066	(2.779-3.353)	2.833	(2.116-3.550)	0.523	0.810
**Algesimeter**	0	11.166	(-13.341-35.674)	10.666	(-0.874-22.207)	16.166	(2.88-29.447)	0.663	0.944
	30	8.166	(1.792-14.540)	12.666	(0.412-24.920)	17.000	(-0.210-34.210)	0.542	0.189
	60	13.833	(-4.603-32.270)	14.833	(-7.707-37.373)	18.000	(-2.894-38.894)	0.365	0.996
	120	15.833	(-4.360-36.027)	3.666	(-1.353-8.686)	16.500	(-1.542-34.542)	0.805	0.233
	180	11.833	(-1.21-24.880)	8.400	(2.55-14.241)	9.500	(3.808-15.191)	0.661	0.104
	240	15.000	(0.675-29.324)	9.033	(1.41-16.650)	14.833	(-7.707-37.373)	0.764	0.245

λ-C: λ-carrageenan; L-CSh: lidocaine - chitosan hydrochloride; EMLA: Eutectic Mixture of Local Anesthetics; CI: confidence interval

Table S5. Paw withdrawal threshold after lidocaine-chitosan-liposomes administration

	Time after λ-C delivery (minutes)	L-CS-LP (n=10)		Control group (n=10)		EMLA Group (n=10)		P Value	
		Mean	95% CI	Mean	95 % CI	Mean	95 % CI	L-CS-LP vs Control	L-CS-LP vs EMLA
**Cold plate**	0	14.000	(11.515-16.484)	7.666	(-2.675-18.009)	3.333	(1.899-4.767)	0.179	0.333
	30	8.666	(1.077-16.255)	8.333	(3.162-13.504)	4.666	(-5.675-15.009)	0.300	0.058
	60	6.000	(-0.572-12.572)	6.333	(-0.837-13.504)	2.333	(-3.403-8.070)	0.788	0.788
	120	2.333	(-1.461-6.127)	5.666	(-2.318-13.652)	3.000	(-5.956-11.956)	0.780	0.942
	180	2.333	(-1.461-6.127)	5.000	(0.697-9.302)	2.666	(-4.922-10.255)	0.879	0.667
	240	1.666	(0.232-3.100)	1.666	(-1.201-4.535)	2.000	(-0.484-4.484)	**0.000**	0.333
**Hot plate**	0	5.766	(3.758-7.774)	4.133	(1.477-6.789)	7.033	(4.377-9.689)	0.272	0.454
	30	5.233	(-1.003-11.470)	3.733	(-1.371-8.838)	2.533	(2.389-2.676)	0.988	0.275
	60	4.366	(4.079-4.653)	5.133	(2.620-7.646)	5.633	(1.257-10.009)	0.698	**0.018**
	120	4.633	(1.429-7.837)	2.933	(0.625-5.241)	4.233	(0.496-7.970)	0.910	0.109
	180	4.233	(2.865-5.601)	3.533	(2.307-4.758)	2.766	(2.049-3.483)	0.235	0.967
	240	5.566	(2.139-8.993)	3.066	(2.779-3.353)	2.833	(2.116-3.550)	0.116	0.551
**Algesimeter**	0	6.000	(2.273-9.726)	10.666	(-0.874-22.207)	16.166	(2.88-29.447)	0.402	0.121
	30	5.500	(1.021-9.978)	12.666	(0.412-24.920)	17.000	(-0.210-34.210)	0.244	0.488
	60	4.600	(-0.386-9.586)	14.833	(-7.707-37.373)	18.000	(-2.894-38.894)	0.693	0.054
	120	8.500	(5.213-11.786)	3.666	(-1.353-8.686)	16.500	(-1.542-34.542)	0.121	0.917
	180	7.166	(1.995-12.337)	8.400	(2.55-14.241)	9.500	(3.808-15.191)	0.569	0.667
	240	7.166	(1.995-12.337)	9.033	(1.41-16.650)	14.833	(-7.707-37.373)	0.863	0.618

λ-C: λ-carrageenan; L-CS-LP: lidocaine-chitosan-liposomes; EMLA: Eutectic Mixture of Local Anesthetics; CI: confidence interval

Table S6. Statistical indicators for the researched substances

	Cold Plate						Hot Plate					
**Time (min.)**	0	30	60	120	180	240	0	30	60	120	180	240
**Lidocaine - Chitosan Hydrochloride**												
Min	6.0	6.0	1.0	3.0	0.0	0.0	3.5	4.2	4.3	3.1	4.1	3.8
Max	11.0	10.0	14.0	12.0	11.0	3.0	6.9	5.5	13.8	6.8	4.9	6.8
SD	2.64	2.30	6.50	4.72	5.85	1.52	1.71	0.70	5.00	1.85	0.40	1.56
**Control**												
Min	3.00	6.00	3.00	2.0	4.00	1.00	2.90	2.40	4.50	2.30	3.20	3.00
Max	11.0	10.0	8.00	8.0	7.00	3.00	4.80	6.10	6.30	4.00	4.10	3.20
SD	4.16	2.08	2.88	3.21	1.73	1.15	1.06	2.05	1.01	0.92	0.49	0.11
**EMLA™**												
Min	3.00	0.00	1.00	0.00	0.00	1.00	6.10	2.50	3.60	2.80	2.60	2.50
Max	4.00	8.00	5.00	7.00	6.00	3.00	8.20	2.60	6.70	5.80	3.10	3.00
SD	0.57	4.16	2.30	3.60	3.05	1.00	1.06	0.05	1.76	1.50	0.28	0.28
**Lidocaine - Cyclodextrin 2.5%**												
Min	12.0	3.00	0.00	3.00	3.00	0.00	3.50	2.20	2.00	1.80	2.10	3.00
Max	12.1	20.0	14.0	11.0	11.0	8.00	5.00	4.10	2.60	2.60	2.50	4.20
SD	0.05	8.88	7.00	4.00	4.35	4.00	0.75	0.96	0.30	0.43	0.20	0.60
**Lidocaine - Chitosan 4%**												
Min	6.00	9.00	1.00	0.00	0.00	1.00	3.60	3.50	3.60	3.80	3.00	3.10
Max	17.0	17.0	5.00	2.00	5.00	4.00	4.20	3.80	4.00	4.60	5.40	5.80
SD	5.85	4.04	2.08	1.00	2.64	1.52	0.32	0.17	0.20	0.41	1.20	1.36
**Lidocaine - Chitosan - Liposomes**												
Min	13.0	6.00	3.00	1.00	1.00	1.00	4.90	2.60	4.30	3.20	3.70	4.00
Max	15.0	12.0	8.00	4.00	4.00	2.00	6.50	7.60	4.50	5.70	4.80	6.60
SD	1.00	3.05	2.64	1.52	1.52	0.57	0.80	2.51	0.11	1.28	0.55	1.37
**Lidocaine – Liposomes 1.7%**												
Min	6.00	2.00	3.00	0.00	0.00	0.00	5.20	2.80	3.70	3.70	3.40	3.30
Max	13.0	10.0	4.00	6.00	2.00	1.00	6.30	4.40	4.70	6.80	6.50	6.50
SD	3.60	4.16	0.57	3.21	1.15	0.57	0.60	0.89	0.51	1.55	1.59	1.61
**Lidocaine**												
Min	12.0	3.00	1.00	0.00	0.00	0.00	4.70	3.30	3.50	3.80	3.80	3.30
Max	20.0	16.0	3.00	2.00	2.00	1.00	5.20	3.90	5.90	6.00	5.10	4.40
SD	4.16	6.55	1.15	1.00	1.00	0.57	0.26	0.30	1.28	1.10	0.70	0.55

Table S7. Statistical indicators for the researched substances

	Algesimeter					
**Time (min.)**	0	30	60	120	180	240
**Lidocaine - Chitosan Hydrochloride**						
Min	4.5	6.0	7.5	9.5	6.5	10.5
Max	22.5	11.0	22.0	25.0	17.0	21.5
SD	9.86	2.56	7.42	8.12	5.25	5.76
**Control**						
Min	7.50	7.00	4.5	2.5	6.0	5.50
Max	16.0	16.0	21.5	6.0	10.7	11.0
SD	4.64	4.93	0.07	2.02	2.35	3.06
**EMLA™**						
Min	11.5	13.0	8.50	9.0	7.50	4.50
Max	22.0	25.0	24.5	23.5	12.0	21.5
SD	5.34	6.92	8.41	7.26	2.29	9.07
**Lidocaine - Cyclodextrin 2.5%**						
Min	5.80	4.50	5.00	10.5	11.0	8.50
Max	21.0	11.5	7.40	15.5	12.4	14.0
SD	7.61	3.68	1.20	2.64	0.73	2.92
**Lidocaine - Chitosan 4%**						
Min	7.50	7.50	6.00	4.50	6.50	4.50
Max	25.0	20.0	25.0	20.0	7.50	6.00
SD	9.69	6.38	9.84	8.54	0.50	0.76
**Lidocaine - Chitosan - Liposomes**						
Min	4.50	4.00	2.30	7.00	5.00	5.50
Max	7.50	7.50	6.00	9.50	6.50	9.50
SD	1.50	1.80	2.00	1.32	0.76	2.08
**Lidocaine – Liposomes 1.7%**						
Min	6.00	5.00	5.50	4.00	8.50	5.50
Max	10.5	14.0	12.5	10.5	12.5	13.5
SD	2.36	4.93	3.54	3.32	2.17	4.16
**Lidocaine**						
Min	5.00	4.00	4.00	3.50	4.00	3.50
Max	8.50	4.50	7.50	5.00	6.00	11.0
SD	1.80	0.28	1.89	0.76	1.04	4.07
